# Deep Tillage Strategies in Perennial Crop Installation: Structural Changes in Contrasting Soil Classes

**DOI:** 10.3390/plants11172255

**Published:** 2022-08-30

**Authors:** Raphael Passaglia Azevedo, Lara Mota Corinto, Devison Souza Peixoto, Tomás De Figueiredo, Gustavo Cesar Dias Silveira, Pedro Maranha Peche, Leila Aparecida Salles Pio, Paulo Humberto Pagliari, Nilton Curi, Bruno Montoani Silva

**Affiliations:** 1 Department of Soil Science, Federal University of Lavras, Av. Doutor Sylvio Menicucci 1001, Lavras CEP 37200-900, MG, Brazil; 2 Mountain Research Center (CIMO), Polytechnic Institute of Bragança (ESA/IPB), Santa Apolonia Campus, 5300-253 Bragança, Portugal; 3 Department of Agriculture, Federal University of Lavras, Av. Doutor Sylvio Menicucci 1001, Lavras CEP 37200-900, MG, Brazil; 4 Southwest Research and Outreach Center, Department of Soil, Water, and Climate, University of Minnesota, 23669 130th St., Lamberton, MN 56152, USA

**Keywords:** deep mixing, subsoiling, soil electrical resistivity, resistance to penetration

## Abstract

Tillage modifies soil structure, which can be demonstrated by changes in the soil’s physical properties, such as penetration resistance (PR) and soil electrical resistivity (*ρ*). The aim of this study was to evaluate the effect of deep tillage strategies on three morphogenetically contrasting soil classes in the establishment of perennial crops regarding geophysical and physical-hydric properties. The experiment was conducted in the state of Minas Gerais, southeastern Brazil. The tillage practices were evaluated in Typic Dystrustept, Rhodic Hapludult, and Rhodic Hapludox soil classes, and are described as follows: MT—plant hole; CT—furrow; SB—subsoiler; DT—rotary hoe tiller; and DT + calcium (Ca) (additional liming). Analyses of PR and electrical resistivity tomography (ERT) were performed during the growing season and measurements were measured in plant rows of each experimental plot. Undisturbed soil samples were collected for analysis of soil bulk density (Bd) at three soil depths (0–0.20, 0.20–0.40, and 0.40–0.60 m) with morphological evaluation of soil structure (VESS). Tukey’s test (*p* < 0.05) for Bd and VESS and Pearson linear correlation analysis between Bd, *ρ*, and PR were performed. Soil class and its intrinsic attributes have an influence on the effect of tillage. The greatest effect on soil structure occurred in the treatments DT and DT + Ca that mixed the soil to a depth of 0.60 m. The *ρ* showed a positive correlation with Bd and with PR, highlighting that ERT may detect changes caused by cultivation practices, although ERT lacks the accuracy of PR. The soil response to different tillage systems and their effects on soil structure were found to be dependent on the soil class.

## 1. Introduction

Soil structure controls many processes in soils [1] therefore, understanding and knowing how to manage it can be beneficial for both agricultural production and the environment. Creating soil conditions favorable to root growth, with consequences on crop development and production, involves optimizing the supply of air, water, and heat from the soil by favoring uptake of water and nutrients [2]. High-density layers (of pedogenetic origin), which normally behave in a manner similar to compacted layers (from human activity), not only increase the risk of water erosion [3,4], but also limit crop growth and development by restricting the root system to the surface layer [5], therefore making the development of deep roots subject to the conditions of pre-existing pores [6] and much more vulnerable to dry conditions during the winter and short droughts during the rainy season, which are very common in Brazilian conditions.

Mechanical modifications in the soil subsurface for the purpose of alleviation of high-density or compacted soil layers are known as deep tillage operations [7]. Deep tillage can increase the soil volume used by crop roots and may thus assist in resolving restrictions in the subsoil, especially by increasing water uptake [7,8,9]. This positive effect can transform marginal areas into productive systems [10,11,12] due to the resulting improvement in soil physical quality, soil function, and the provision of ecosystem services [13].

The research our team has been conducting over the last decade has resulted in positive results with the use of deep tillage, especially in non-irrigated coffee [14]. The success of this system of management is due to the greater deepening of crop roots, allowing greater uptake of water from deep layers, resulting in higher yields and longevity of the coffee fields grown in areas of Cambissolos (Inceptisols) and Latossolos (Oxisols) [9,11,15,16,17,18,19,20,21,22,23]. However, from a soil physical environment perspective, the question arises as to whether the same soil management system will lead to similar results in different soil classes. Limited research data are available on the performance of deep tillage in soils with contrasting physical properties from tropical regions under perennial crop cultivation [24,25]. Most of the peer-reviewed studies on deep tillage have been performed on annual/cereal crops [7] and/or these studies have not been performed on soil classes with contrasting structure [24,25]. No work has been found to evaluate the effect of tillage systems on soil classes with contrasting structures.

Changes in soil structure caused by tillage can affect soil chemical and physical-hydric properties, such as soil moisture, soil solution ionic concentration, soil porosity, and bulk density, which leads to variations in soil electrical resistivity (*ρ*) values [26,27,28,29,30,31]. Jeřábek et al. [32] and Piccoli et al. [33] used Electrical Resistivity Tomography (ERT) to identify the depth of soil “hardpans”, which then confirmed the data obtained by resistance to penetration and bulk density. Roodposhti et al. [34] discussed how *ρ* varies in accordance with soil moisture and bulk density, showing that at the same moisture content, increases in bulk density result in a reduction in *ρ*. The same was reported by Melo et al. [35], who observed an inverse relationship between *ρ* and bulk density. However, this relationship is inverted for soils with moisture contents greater than 18 cm^3^ cm^−3^. In this respect, ERT is considered one of the most effective geophysical methods for agricultural and environmental studies [36]. In addition, it is a fast and non-invasive method that is able to monitor the spatial and temporal variability of many soil chemical and physical properties (structure, moisture, fluid composition) [33,37,38,39,40].

Thus, the hypotheses tested in this study were that (i) ERT, alone or in combination with other physical attributes, is able to show the efficiency of deep tillage methods in contrasting soil classes regarding structure; and, (ii) soils that have higher natural bulk density have a better response to deep mixing and exhibit greater structural alleviation. Our expectation is to find correlations between *ρ* and the other physical properties that will confirm the potential of ERT under field conditions. Therefore, the aims of this study were to (i) evaluate the effects of deep tillage systems on the establishment of a perennial crop related to the structural quality of three contrasting soil classes and intrinsic attributes; and, (ii) determine the changes in electrical resistivity caused by soil tillage, as well as to correlate *ρ* with soil physical properties.

## 2. Results and Discussion

### 2.1. Penetration Resistance (PR)

**Figure 1:**
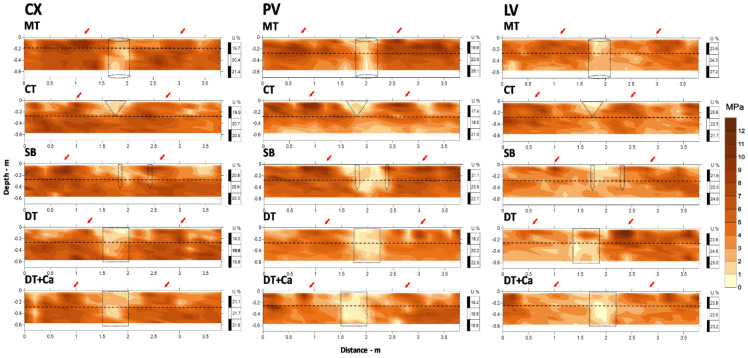
2D maps of soil resistance to penetration for three soil classes (CX, PV, and LV) under five different soil tillage treatments [MT: minimum tillage with a pit (0.4 m in diameter by 0.7 m deep); CT: conventional tillage (0.25 m deep); SB: subsoiler with booted ripper points on two shanks (0.45 m deep); DT: deep mixing tillage with rotary hoe tiller (0.5 wide by 0.6 m deep); DT + Ca: deep mixing tillage with rotary hoe tiller (0.5 wide by 0.6 m deep) + additional liming. The horizontal dashed line demarcates the working depth of the plow (approximately 0.25 m); dotted lines project the working area of the implements used; red arrows indicate the signs of machine traffic; U: soil moisture based on the weight (U, %) of each layer at the time of data collection. Measurements were performed eight months after tillage operations.

The results of PR for the Typic Dystrustept (CX), Rhodic Hapludult (PV) and Rhodic Hapludox (LV) soil classes under the different tillage treatments (minimal tillage—MT, conventional—CT, subsoiler—SB, deep mixing tillage—DT, and deep mixing tillage with added supplementary liming—DT + Ca) are shown in the form of 2D maps in Figure 1. Figure 1 presents values of soil resistance to penetration (MPa) measured in a transect perpendicular to the plant row to highlight the effect of tillage. PR is an able and effective tool for accessing the physical status of the soil or a sequence of changes in soil structure [40,41,42,43] and was used for the presentation, discussion and comparison of results regarding the efficiency of the tillage methods, with a limit value for root development of 3 MPa [44,45].

The effect of the different soil tillage systems that caused reductions in PR was clearly observed in all the soil classes and treatments, shown in the maps by light-colored centralized zones (RP less than 3 MPa). Dotted lines indicate the expected projection of the working area of each implement, which helps in perceiving the differences in the efficiency of tillage systems in each soil class, indicated by the PR values. In general, PR values less than 3 MPa were found between 1.6 and 2.0 m on the “x” axis (plant row) and vary in depth with the action of each implement, whereas the higher values (greater than 3 MPa) did not have a defined pattern of distribution, but were affected by the soil structure itself at depths where there was no effect of tillage (below 0.25 m) and near the surface (0–0.10 m), coinciding with the signs of machine traffic (Figure 1—red arrows). It should be noted that all the machine traffic locations could not be monitored due to the difficulty of controlling the machine and implementing traffic within the experimental area (Figure 1).

In relation to soil class, in general, the mean of PR throughout the sector was the lowest for LV—3.90 MPa, followed by CX—5.14 MPa and PV—5.55 MPa, respectively. Observation of the effect of the treatment in its working area, considering the distance of 1.5–2.5 m and depth that varies in accordance with each implement (MT—0.6 m, CT—0.25 m, SB—0.45 m, DT—0.60 m, and DT + Ca—0.60 m), showed that even 8 months after the implantation of soil tillage, the effects caused by it were clear, above all in PV, where there was a greater contrast of the PR values between the areas with tillage and without tillage, followed by CX and then by LV, where the contrast was less.

In the CX soil class, the greatest tillage efficiency was achieved by MT and CT, which achieved 100% to the depth desired, with PR values less than or equal to 3 MPa, whereas the effect of the other treatments did not reach the depth expected (approximately 90%). This can be observed comparing the effect of tillage on MPa to the dotted lines that project the expected result. The efficiency of the implements in MT and CT may have been favored by the previous action of plowing and disking.

Under natural conditions, this soil (CX) exhibits physical restriction starting at 0.05 m, with reduction in the amount of sand and a doubling in the amount of silt fraction compared to the previous layer (0–0.05 m), favoring an increase in Bd (Table 1). This shallow physical restriction is an intrinsic property to most of the soils included in this class [46]. This may have led to an increase in mechanical resistance of the soil at the time of tillage. However, deep tillage aims at alleviating the high soil density observed in this layer for the purpose of improving the physical quality of the soil for crops [9]. Much of the references cited here [9,11,16,22] used soil tillage methods similar to those used in the present study in setting up coffee fields. Improvements in soil physical quality at depth one year after the implantation of the crop are reported and likely due to an increase in aeration capacity and in water availability in the Typic Dystrustept (Cambissolo Háplico distrófico típico) under study. In the PV and LV soil classes, all the treatments had PR values less than or equal to 3 MPa in the working areas of the tillage implements. The contrast of the PR values within and outside the plant row, especially in CX and PV in comparison with LV, is noteworthy (Figure 1).

CX and PV are soil classes with similar characteristics regarding high natural density of horizons in the subsurface (B horizon), which explains the high values of PR. Cambissolos (Inceptisols) generally have physical conditions that are not favorable to plant development, mainly due to the greater silt in relation to clay fraction content that characterizes these less weathered soils. They are characterized by the subangular block structure in the B horizon and coherent massive structure in the C horizon, which have a direct effect on their naturally high density [46], and this results in low soil porosity. Furthermore, the reduced thickness of the solum (A + B horizons) of the CX in this study is noteworthy (Table 1). Argissolos (Ultisols in this study), however, have mature pedogenesis, promoting accumulation of clay fraction and occurrence of clay skins in the textural B diagnostic horizon [50], where the block structure predominates. Latossolos (Oxisols), for their part, have an advanced stage of weathering-leaching and are deep soils [50]. Due to its greater gibbsite content, this soil class shows granular structure, which promotes high macroporosity [51] and lower bulk density compared to CX and PV (Table 1), which explains the lower values of PR observed in Figure 1.

The results of this study indicate that the tillage systems were efficient in reducing the PR of the soils, notably within the working depth of the implements. This suggests that even soil classes that have naturally greater restriction for agricultural use, such as CX [46,52] and PV, if properly tilled, can show physical improvement, with PR values that do not limit crop growth (less than 3 MPa). Nevertheless, Figure 1 shows that the effectiveness of tillage systems may have been affected by the soil intrinsic attributes, both those related to mechanical resistance to penetration and those pertinent to natural reconsolidation of the soil [53,54].

Several studies [40,41,42,43] have suggested the development of a soil tillage efficiency indicator. Thus, we propose that the tillage systems efficiency be evaluated by comparing the areas/depths of the soil under mechanical intervention with lateral areas/depths not subjected to management practices. The indication criteria should also include reduction of PR in relation to the area not under management (considering a critical value for the crop or not) and the depth and width of the working area of the tillage system visualized by the 2D map of PR.

### 2.2. Electrical Resistivity Tomography (ERT)

**Figure 2:**
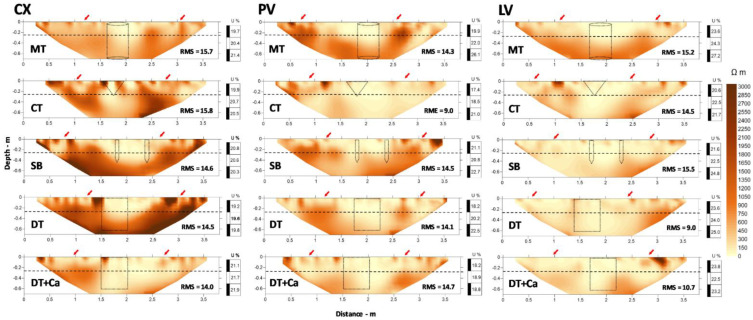
2D maps of soil electrical resistivity for three soil classes (CX, PV, and LV) under five different soil tillage treatments [MT: minimum tillage with a pit (0.4 m in diameter by 0.7 m deep); CT: conventional tillage (0.25 m deep); SB: subsoiler with booted ripper points on two shanks (0.45 m deep); DT: deep mixing tillage with rotary hoe tiller (0.5 wide by 0.6 m deep); DT + Ca: deep mixing tillage with rotary hoe tiller (0.5 wide by 0.6 m deep) + additional liming. The horizontal dashed line demarcates the working depth of the plow (approximately 0.25 m); dotted lines project the working area of the implements used in each tillage treatment; U soil moisture based on the weight (U, %) of each layer at the time of data collection. Measurements were performed eight months after tillage operations.

Similar to PR, *ρ* values shown in the form of 2D maps (Figure 2), exhibited strong spatial variability in the transects of each treatment in the three soil classes. The quality of the *ρ* maps can be observed by the values in the lower right corner, which indicate the root mean square (RMS) of each treatment in each soil class after inversion (Figure 2). Variation in the RMS ranged from 9% to 15.8%, with a mean of 13.7% (Table 2).

In evaluation of soil structure after management practices, [26] observed RMS less than 10% after 4 to 6 interactions, just as in [28], with RMS lower than 9%. However, when there is the possibility of a greater number of interactions, it is possible to achieve lower RMS, as in [55], who observed RMS varying from 1.20 to 1.70% for interactions ranging from 6 to 7. In the same way, [36], studying the root-soil interaction under irrigation systems, stipulated a value for RMS of 16%, just as [56], when analyzing the water dynamics in the subsurface directed by land use, reported an RMS of 15.75% in their maps.

The values of RMS found in our study are greater than those found in studies conducted in soils of temperate regions [26,28,36,55,56], which indicates the need for more studies involving soils of tropical regions. The greater error values in evaluations of ERT can be explained by the greater difficulty in establishing good contact between the electrodes and the dry soil, which affects electrical readings and the accuracy of inversion of resistivity [57,58]. In addition, soils with greater clay content, specially those having high-activity clays, can form cracks during dry periods [59], producing noise that increases errors in readings. Abrupt changes in soil structure are another factor that can be source of noise and lead to an increase in RMS, especially when associated to changes in porosity caused by soil management.

The *ρ* values ranged from 7 to 7000 Ωm; however, *ρ* values up to 3000 Ωm were used for creating the maps, understanding that values greater than that would pollute and could impede accurate interpretation of the images. This range of values can be considered high compared to that reported in the literature with similar objectives: 30–1680 Ωm [30], 150–1061 Ωm [55], 20–80 Ωm [32], and 0–500 Ωm [33]. Yet, all of these were in soils of temperate regions with no research reporting results for soils under tropical conditions. The highest amplitude of *ρ* values was observed in CX for the treatment with the subsoiler—SB (0.45 m depth), followed by PV also in the SB treatment, and LV in DT + Ca (Figure 2).

For all the soil classes and treatments analyzed, there as a tendency for an increase in *ρ* with an increase in depth, proceeding from less dense areas to denser ones. At depths where soil tillage occurred^,^ from 1.5–2.5 m, approximately (x axis, Figure 2) and thus regions of greater structural alleviation (blue dotted lines), the lowest *ρ* values were observed (*ρ* less than 300 Ωm). In CX, it is possible to identify clear patterns of the effect of deep tillage, specially in the SB and DT treatments compared to the other treatments. The results of this study show that *ρ* maps can provide a better estimate of horizontal action as a function of implement being used than those obtained with PR maps (Figure 1). In PV, similar patterns to CX were observed, but were not so easily separated out from the background soil conditions. In contrast, in LV, it was not possible to distinguish clear patterns among any of the deep tillage treatments.

The greatest *ρ* values are in the non-tilled areas, especially below 0.25 m, the mean depth reached by the plow, and in some small areas near the surface, to approximately 0.10 m (Figure 2). Besson et al. [26] observed lower *ρ* values in machine traffic areas, with greater Bd, than in non-compacted areas, without, however, determining the exact position of the wheel tracks. In our study, the distribution of *ρ* was consistent with the values of PR except for the surface PR. The red arrows in Figure 1 demarcate high PR areas, which we indicated as signs of machine traffic. Our results showed that the ERT was unable to demarcate these same positions.

Changes in *ρ* under the conditions evaluated may largely be caused by changes in bulk density, moisture, and porosity [28,31]. Temperature variations may affect *ρ* values [26,29,38], and may even hinder the ability of data collection in the field [56]. However, for this study, which was performed under tropical conditions, we assumed that the temperature was stable during acquisition of *ρ* [26,60]. Zhou et al. [61] showed that the greatest changes in *ρ* take place at around and below 0 °C. In addition, another source of variation in *ρ* is the effect of integration of the hemisphere, which means that the results of *ρ* are affected by characteristics lateral to the ERT transect [27]. Variations in moisture also create difficulties in interpretation of the results [32,35,62].

Areas of greatest *ρ* below the depth of 0.25 m, especially in CX and PV, are consistent with the results of PR and with the morphological attributes of these soil classes that have naturally greater bulk density in the subsurface [46]. The ERT was able to identify changes caused by the different soil tillage methods, but it was unable to precisely demarcate the region changed by each tillage method. Studies performed for the purpose of detecting structural changes in agricultural areas show that ERT does not have sensitivity to abrupt variations in *ρ*, such as rock fragments or even high-density clods surrounded by material of high porosity [27,30]. In addition, soil moisture has a predominant effect on *ρ* in detriment to structural changes [28,34,63]. Melo et al. [35] simultaneously studied the relationship among *ρ*, moisture content in the soil, and the degree of compaction in a Brazilian Typic Hapludox [64] and concluded that soil moisture content has a greater effect on *ρ* than the degree of compaction. It should be emphasized that 2D resistivity tomography was performed in the driest season of the year, winter, in which the last rainfall of 7 mm occurred 10 days before data collection (ERT and PR). In that period, the mean maximum temperature was 24 °C and the mean minimum was 10 °C, with relative humidity of 63.5%, which led to soil moisture below FC (Table 1, Figure 2).

Seladji et al. [62] observed that the relationship between bulk density and *ρ* is controlled by soil moisture, and is significant and negative when less than 0.25 g g^−1^ for French soils. Studies performed by Melo et al. [35] show that there is an inversion of interpretation of the relationship between bulk density and *ρ* with variation in moisture, specifically at the value of 0.18 m^3^ m^−3^, or approximately 13.4% moisture, based on weight in a Typic Hapludox [64] in the same region as this study, whereas most studies show a direct relationship between *ρ* values and soil porosity [32,38,65,66] under dry soil conditions. Melo et al. [35] showed that above this soil moisture content (18 m^3^ m^−3^), the relationship is reversed between *ρ* and porosity. Results of this nature are presented by Naderi-Boldaji et al. [67] and Piccoli et al. [33], who identified areas of alleviation of porosity by plowing areas of low *ρ*, whereas a compacted area (plow pan) was identified by high *ρ*.

The soil profile evaluated by the ERT may act as an insulating or conductive material depending on its moisture content. Moisture content is the main factor that controls *ρ* because the main mechanism responsible for conduction of electrical current in the soil is electrolysis [38]. Under high moisture conditions, the soil matrix will act as a resistive material and the water present in the pores as a conductor, whereas under low moisture, the air present in the pores leads to an increase in resistivity, and the soil matrix will be a conductor [35]. Therefore, under the conditions of increase in bulk density and high moisture, there is a reduction in the amount of water stored due to the reduction in porosity. As a result, there is a greater amount of resistive material (soil matrix) and consequently high *ρ* in compacted locations [67]. In contrast, low *ρ* in porous areas suggests that the porous spaces may be filled with water, which increases conductivity. This mechanism presumably explains the low *ρ* values that predominate in the LV (Figure 2).

### 2.3. Bulk Density (Bd)

**Figure 3:**
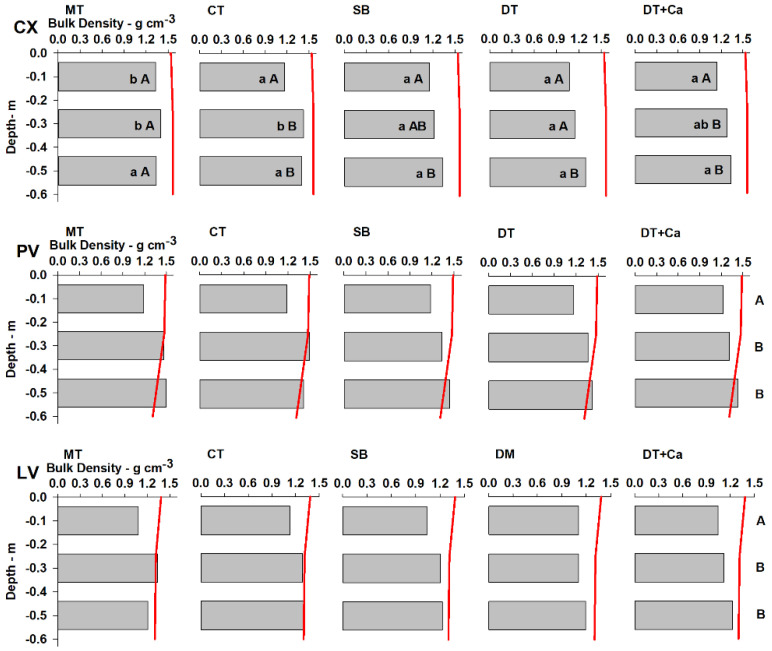
Bulk density (Bd) and critical bulk density (vertical red line) for three soil classes under different soil tillage systems for a perennial crop. Measurements were performed seven months after tillage operations. Mean values followed by the same lowercase letters in the row and uppercase letters in the column do not differ from each other by Tukey’s test (*p* < 0.05). MT: minimum tillage with a pit (0.4 m in diameter by 0.7 m deep); CT: conventional tillage (0.25 m deep); SB: subsoiler with booted ripper points on two shanks (0.45 m deep); DT: deep mixing tillage with rotary hoe tiller (0.5 wide by 0.6 m deep); DT + Ca: deep mixing tillage with rotary hoe tiller (0.5 wide by 0.6 m deep) + additional liming; Critical bulk density by according to [72]; (CX: Typic Dystrustept, PV: Rhodic Hapludult, and LV: Rhodic Hapludox).

The results of the analysis of Bd carried out on the soil classes PV, CX, and LV under different tillage systems (MT, CT, SB, DT, and DT + Ca) are shown in Figure 3, except for MT, because sample collection performed within the plant row would cause considerable damage to the plant. The soil tillage strategies affected Bd for the three soils in a different way. In CX there was a significant effect of the different tillage systems, which did not occur for PV and LV; nevertheless, there were differences among the depths for the three soil classes (Figure 3). In the PV soil, unlike the others, at the 0.20–0.40 m depth, all the treatments exceed the critical bulk density limit, 1.32 and 1.30 g·cm^−3^, 0.20–0.40 and 0.40–0.60 m, respectively (vertical red line–Figure 3), suggesting that its subsoil environment is more restrictive to plants. However, the use of treatments labeled “deep tillage” (DT and DT + Ca) reduced the Bd in relation to Bdc, especially compared to MT and CT treatments in the PV and LV soils. This result shows how effective soil tillage was in loosening the soil and physically improving the root environment. Few studies relate the morphological attributes of soil classes to plant growth or to management practices. Studies generally relate the soil physical properties to management practices only in the arable layer, 0–0.30 m on average, and do not consider the morphological attributes of each horizon in discussion or their effect on crop growth and productivity [68,69,70,71].

In PV and LV soils in general, the Bd increased with depth, without difference between the 0.20–0.40 m and 0.40–0.60 m layers. Lower Bd in the 0–0.20 m layer is associated with its greater organic matter content (Table 1). The absence of effect among the tillage systems on the Bd for the PV and LV soils can be explained by the lower sensitivity of Bd in detecting changes in structural alleviation. In addition to lower sensitivity of Bd, lower persistence of the effects of tillage in PV and LV is related to faster reconsolidation in these soils than in CX. Reconsolidation or the “age-hardening” phenomenon [73,74,75] is a natural process that occurs in a slower way at depth than on the surface and mainly depends on accumulated rainfall [53,76] and wetting and drying cycles [77], even without machine traffic [78]. This phenomenon is the result of the rearrangement of soil particles, especially clay particles, in new positions of minimum free energy and strengthening of the cementation bonds in new points of contact between pairs of mineral particles [73]. This can explain why CX has slower reconsolidation, since PV and LV have greater clay in relation to silt content, a higher degree of weathering-leaching, and greater concentration of Fe and Al oxides, which, for their part, accelerate such process [79].

Corroborating the fact that the effects of soil management in this study are not perceptible through Bd at 9 months after tillage, Reichert et al. [80] report that in a subtropical Argissolo (Ultisol), in less than one year, the effects of chisel plowing observed through changes in Bd disappeared, reaching a compaction condition similar to that of an area under a no-tillage system for 10 years. The authors attributed this effect to reconsolidation of the soil, due to mechanical disruption of the aggregates and their later rearrangement. Loss of the effect of soil management is also observed in Drescher et al. [81] through Bd in a period shorter than a year in a clayey Latossolo Vermelho (Oxisol). Nicoloso et al. [82] evaluated the effect of mechanical chisel plowing on a very clayey Latossolo (Oxisol) and noted a short-term effect of the practice, without obtaining improvement in the physical conditions nine months after the operation. Scarpare et al. [83] evaluated deep tillage similar to the DT and DT + Ca treatments in a sandy-clay kaolinitic Latossolo (Oxisol) and observed reduction in Bd and increases in the density of the root system in soils under sugarcane cropping. However, the effect of this tillage on Bd persisted for one year, but was not observed in the second year. Those results differ especially from the results in our study, mainly due to variation in texture and mineralogy between the Latossolos (Oxisols). Bavoso et al. [84] evaluated the quality and the resilience of two Latossolos (Oxisols) and found that the soil with greater clay content had greater resilience. Peixoto et al. [85] used a machine learning algorithm to rank soil properties that are more sensitive in detecting structural changes due to soil tillage: PR was most important and Bd appeared in sixth place. This is in agreement with a previous study of Abreu et al. [86], who also showed greater sensitivity of PR in relation to Bd. In this respect, Simões et al. [76], evaluating the effect of subsoiling on an Ultisol on the east coast of North America, showed, through PR, evident reconsolidation in this soil class at 11 months after tillage. This effect is expected; as reported by Theadgill [40], for Ultisol, reconsolidation of greater density layers generally occurs in one year. Thus, Busscher et al. [41] observed loss of the effect of subsoiling in an Ultisol, with reconsolidation of around 75% in one year and 90% in two years. However, reconsolidation cannot clearly be observed in this study using PR (Figure 1).

In CX, the lower values of Bd are associated with mobilization of the soil by tillage, especially in the 0–0.20 m depth. In the other depths, Bd is consistent with the working depth of the implements. The Bd values of the surface layer (0–0.20 m) show that the effect of plowing and disking associated with the other implements led to significant reduction in Bd in relation to the MT treatment, where mechanical mobilization did not occur, just as in the subsequent layer, where SB, DT, and DT + Ca led to reduction in Bd compared to MT and CT. However, in the 0.40–0.60 m layer, the Bd values are not different, which corroborates the fact, described above, that it is possible that in the CX, the effective depth of the implement was not reached (Figure 1). The shallowest C horizon, with coherent massive structure, helps to understand such effect. These results are can be used to show that deep tillage is important in reducing Bd and in structural alleviation of higher density soils. This beneficial effect was found in subsoiled soils in Germany [87,88] and in Brazilian Cambisols (Inceptisols) with the DT treatment [9,11,15,16] as long as the clay:silt ratio is greater than 0.3, as in the case of the present study (lowest ratio = 1.0). In soils with clay:silt ratios below 0.3, subsoiling has resulted in a collapse of the soil structure and in compaction [7].

Therefore, the lower sensitivity of the Bd measurement in detecting changes brought about by tillage in soils with more advanced weathering-leaching processes and deeper *solum* (Figure 3, PV and LV) can be explained by the factors that act on reconsolidation. The response of the soil in returning to its state before tillage is affected by the organic matter content, texture, and mineralogy [79], as well as climate and weathering [84]. LV and PV have greater clay and Fe and Al oxides content than CX (Table 1 and Table 2) and under environmental conditions of high rainfall, this leads to greater ability of rearrangement of particles, or reconsolidation [53]. Thus, CX, which is less weathered-leached, exhibited a longer duration of the effects of tillage, i.e., it had lower reconsolidation or resilience capacity. Data compiled by Oliveira et al. [21] showed results in tropical Inceptisols and Oxisols (Cambissolos and Latossolos), i.e., porosity modifications may indicate improvements caused by soil tillage that remain up to five years after setting up the coffee crop. This indicates that Bd may not be a good indicator for making inferences regarding soil quality in accordance with the tillage treatment used.

### 2.4. Correlation between ρ and Other Soil Properties

**Figure 4:**
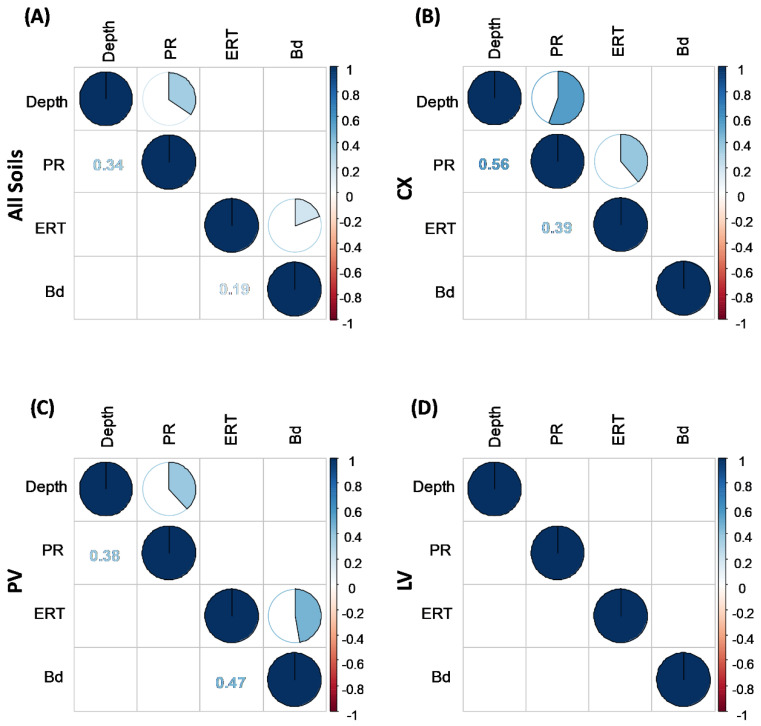
Representations of Pearson linear correlation coefficient for variables of sampling depth, penetration resistance (PR), soil electrical resistivity (RES), and bulk density (Bd), with data from all the soils evaluated (CX: Typic Dystrustept, PV: Rhodic Hapludult, and LV: Rhodic Hapludox) (**A**), and separately for each soil CX (**B**), PV (**C**), and LV (**D**). Only significant correlations are shown (*p* < 0.05); blue circles indicate positive correlations and red circles negative correlations; the upper off-diagonal entry denotes the pie chart for the pairwise correlation level and the lower off-diagonal entry denotes the corresponding pairwise correlation coefficient.

Pearson correlation analyses are presented in Figure 4, which shows only those with significant correlations. Analyzing our entire dataset obtained from the three soil classes, we can observe positive correlations between PR and depth (r = 0.34) and between *ρ* and Bd (r = 0.19) (Figure 4A). Observed in that way, these results are diluted by the three soil classes. For that reason, we opted to separate the correlations by soil class, emphasizing the individuality of each one in its interactions. Thus, we can note an increase in the sensitivity of the analysis, observed by the increase in the correlation coefficient (r) in CX and PV (Figure 4B,C, respectively) by the emergence of significant correlations not otherwise perceived (Figure 4B) and the absence of significant correlations in LV. The lack of significant correlations in LV may be related to its greater resilience, i.e., its rapid ability to restructure itself [79,82,84,89] due to its high flocculating power induced by Al and Fe oxide minerals (gibbsite, goethite, and hematite) that favor the granular structure [51].

In observing *ρ* and its interdependence on the other soil attributes evaluated in this study, Figure 4 shows that *ρ* was affected by the physical properties of each soil class. In general, PR can be used to clearly distinguish the different soil tillage systems and their effects on PR [42,90,91]. In addition, according to Abreu et al. [86] and Peixoto et al. [85,92], PR is more sensitive than Bd in detecting compaction. PR and Bd positively correlated with *ρ*, r = 0.39 for PR in CX, and r = 0.47 for Bd in PV. These correlations indicate the capacity and the sensitivity of ERT as a tool to identify structural changes brought about by management systems in tropical soils, becoming an important alternative for studying temporal and spatial changes in a non-destructive manner, as previously observed by Piccoli et al. [33] in soils in Italy.

The correlation between *ρ* and Bd as found in the literature is negative [26,31,32,93], but also positive, as reported by Naderi-Boldaji et al. [67] and Piccoli et al. [33], which was already discussed above and explained by the effects of high soil moisture [35]. In regard to PR, Piccoli et al. [33] showed a positive correlation with *ρ*, whereas Jeřábek et al. [32] reported a negative correlation. The distribution of clay, silt, and sand fractions among the soil classes (CX, PV, and LV) were different and varied with depth (Table 1), and it appears that the effect of management practices may have superimposed some tendencies of correlation between *ρ* and texture (sand, silt, and/or clay). In contrast, Piccolli et al. [33] showed that the results of *ρ* were driven mainly by texture, *ρ*, and clay: r = 0.30, which was not observed in the present study.

The absence of correlation between PR and Bd can be explained by the marked effect of soil moisture on PR, which did not occur for Bd [33,94]. The correlation between PR and depth in CX and PV can be explained by the morphological attributes of these soil classes, which tend to have naturally higher density in subsurface horizons [41,50,91,95]. Both classes have a block structure (surface-surface contact) in the B horizon, observed during field work. Furthermore, in CX, in addition to the high values of Bd that increase with depth (Figure 3), the main factor that confers higher density is the increase in silt content below 0.05 m (Table 1) [52], sealing the pore system, with a consequent increase in PR (r = 0.56). In a different manner, in PV, the main attributes that caused higher Bd (Figure 3) are the increase in clay content and the presence of clay skins in the Bt horizon (Table 1). In addition, reduction in SOM in the subsurface layers increases PR, as well as Bd (Figure 3) [74]. Unlike CX and PV, LV has an advanced degree of weathering–leaching, with a deep and more homogeneous soil profile [50] due to its greater gibbsite content, which confers good aggregation due to its very small and stable granular structure [51]. For that reason, in LV, the changes caused by management practices are less intense than genetic control of the soil structure, i.e., the soil management practices are not able to supersede the physical effect of the granular structure.

Comparing the effects of the tillage systems obtained by the different tools tested in this study (PR, Bd, and ESS) with the results achieved by ERT, we can affirm that the hypothesis of this study was partially confirmed, since ERT is sensitive to structural changes in the soil, but shows reduced accuracy in demarcating the modified area [27].

## 3. Materials and Methods

### 3.1. Characterization of the Study Area

The present study was conducted in the experimental area of the fruit growing sector of the Universidade Federal de Lavras (UFLA) in the Southeast region of Brazil. The climate in the region is Cwa according to the Köppen climate classification system [96], with hot and humid summers, cool and dry winters, mean annual temperature of 21.6 °C, and mean annual rainfall of 1339.5 mm, concentrated in the months of November to March, according to the data obtained in the period from 01/01/1998 to 01/01/2018 [97].

Due to geological complexity, there is great diversity of the parent material of the soils in the municipality of Lavras [98,99,100]. The campus of UFLA is in the Planalto Atlântico geomorphological unit, under the main influence of leucocratic granitic gneiss (LgG) and mesocratic granitic gneiss (MgG), and there are a wide variety of soil classes even in small areas. This allowed the selection of three classes of soil, classified by mapped by Curi et al. [101], according to Santos et al. [50] and US Soil Taxonomy [64]: Cambissolo Háplico Tb distrófico típico or Typic Dystrustept—CX; Argissolo Vermelho distrófico típico or Rhodic Hapludult—PV; and Latossolo Vermelho distrófico típico or Rhodic Hapludox—LV. These soils are well represented on a national (Brazil) scale (Latossolos—31.6%, Argissolos—26.9%, and Cambissolos—5.26%, Santos et al., [102]), and even on a worldwide scale when considering correlations with the orders in the US Soil Taxonomy classification system (Inceptisols—15%, Ultisols—8%, and Oxisols—8% [103]. The naturally high bulk density found in Inceptisols (Cambissolos) and Ultisols (Argissolos) is a prominent characteristic in these soil classes [41,46], as well as the very small granular structure present in Oxisols (Latossolos) of tropical regions [51]. Both these attributes contribute to the contrasting conditions found in these soil classes for crop growth in regard to root development and soil water availability.

Before the experiment was set up, undisturbed soil samples were collected from the soil horizons for physical characterization (Table 1), and disturbed samples for chemical characterization were collected at the depths of 0–0.2, 0.2–0.4, and 0.4–0.6 m (Table 3), according to standard methods described in Teixeira et al. [47]. The study areas were under fallow conditions for at least 5 years before the experiment was set up. In all three areas, weed control was carried out sporadically with use of herbicide or mechanical cutting. Each area comprises approximately 1200 m^2^.

### 3.2. Experimental Design and Treatments

The same experiment with five treatments and five replications was set up in the three soil classes in a completely randomized design, totalizing 75 experimental plots in the field. Each plot was composed of a plant row with six plants, occupying an area of 40.5 m^2^. The choice of treatments was based on the main soil tillage systems used for establishing perennial crops in Brazil and on works developed by our research group [9,11,15,16,23]. The treatments included: MT—minimum tillage, without soil plowing, and surface furrow (0.10 m depth) for marking the plant row using a furrow opener + plant hole (0.40 m diameter by 0.70 m depth) using a soil borer auger; CT—conventional tillage, disk plowing (0.25 m) + two diskings (0.20 m) + furrow (0.25 m) using a furrow opener; SB—subsoiling, plowing (0.25 m) + two diskings (0.20 m) + subsoiler with booted ripper points on two shanks spaced at 0.50 m (0.45 m); DT—deep mixing tillage, disk plowing (0.25 m) + two diskings (0.20 m) + rotary hoe tiller (0.50 width by 0.60 m depth); DT + Ca—deep mixing tillage and supplementary liming, plowing (0.25 m) + two diskings (0.20 m) + rotary hoe tiller (0.50 width by 0.60 m depth) + additional liming at the depth of 0.40 to 0.60 m to reach 70% and 15% base saturation with Ca and Mg, respectively. For that purpose, liming was applied in the amounts of 3.91, 4.09, and 4.04 t ha^−1^ in the soils CX, PV, and LV, respectively. The rotary hoe tiller is a type of modified rotary hoe with a width of activity of 0.5 m, conducted in strips, composed of a vertical revolving tilling wheel that mixes the surface and subsurface horizons (BigMix^®^ model AS-2, manufactured by Mafes Agromecânica) [104]. The area was tilled in the spring, on 29 November 2018, three days after the last rainfall of 20 mm.

Atemóia (*Annona cherimola* × *Annona squamosa*) was planted on 14 December 2018 at a spacing of 4.5 × 1.5 m, with 6 plants per plot. Soil amendment was performed with liming at the depth of 0 to 0.4 m to raise Ca and Mg saturation to 70% and 15% base saturation, respectively, except for MT, in which liming was performed in the plant hole. In addition, Braquiária Ruziziensis grass (*Urochloa ruziziensis*) was sown between rows and was periodically cut. Liming and fertilization recommendations were according to crop needs, as described in Rozane and Natale [105]. In this study, the effects of addition of complementary liming were only discussed for the DT treatment, since this study focused on analyzing the physical effects on the soil brought about by the different forms of deep tillage.

### 3.3. Variables Analyzed

#### 3.3.1. Electrical Resistivity Tomography (ERT)

Electrical resistivity tomography (ERT) was used to determine the apparent electrical resistivity (*ρa*) of the soil in the field. The readings of *ρa* were obtained with a resistivity meter, model X5tal (Alto Energia, Belo Horizonte, Minas Gerais, Brazil), eight months after soil tillage. The Dipole–Dipole array was used because it has greater horizontal resolution [38] and it is useful for accessing morphological changes in the soil due to tillage [30,32]. In one plot of each treatment in each soil, the readings of *ρa* were taken in transects measuring 3.80 m, perpendicular to the plant row, between the 4th and 5th plant. The transect was composed of 21 rods (electrodes) at a distance of 0.19 m from each other, reaching a depth of 0.70 m, composed of 14 levels at a distance of every 0.05 m, with the first level at 0.09 m from the surface. Each transect in this arrangement resulted in 161 readings (*n* = 161), and a total of 2415 *ρa* values were measured.

The data obtained through ERT compose a 2D section of apparent resistivity (*ρa*) for each treatment. Since they are not homogeneous soils, they do not contain an isotropic current distribution [38], and to resolve this modeling problem, the data were inverted using the Res2DINVDemo 4.9.17 software (Geotomo Software, Penang, Malaysia) [106] for obtaining true resistivity values (*ρ*). All the inversions of the ERT converged with 4 interactions, given the limitations imposed by the Demo version of the Res2DINV program. For that reason, to reduce the noise and the root mean square (RMS), the “RMS error statistic” tool was used, which allows points with greater error or outliers to be excluded. We adopted a maximum error level of 16% for the following reasons: (i) weak signal/noise ratio in the dipole–dipole arrangement, especially when there are wide separations between pairs of current and potential electrodes [107] and (ii) dry soils may manifest cracks due to contraction movement [59], and this may produce anomalies in the current signal, hindering the readings.

The inversion method applied was the smoothness-constrained method, using the mathematical Gauss–Newton least squares method [108]. Of the 161 points of apparent *ρ*, an average of 151 were used for the inversion and construction of the models that generated the maps.

After each measurement of ERT and PR, soil samples were collected along the transect at the depths of 0–0.2, 0.2–0.4, and 0.4–0.6 m for determination of soil moisture by the laboratory oven method.

#### 3.3.2. Penetration Resistance in the Field (PR)

Soil mechanical resistance to penetration (PR) in the field was measured by the cone index using an impact dynamic penetrometer (IAA/PLANALSUCAR-STOLF) with a cone tip at a 30° angle and basal diameter of 1.28 cm [94,109], eight months after soil tillage. The dimensions of the cone tip are in accordance with Standard S313.3 of the American Society of Agricultural and Biological Engineers [110]. Evaluation of PR was considered as reference for comparison with the *ρ* analysis. For that purpose, in each soil in the same plot of each treatment in which *ρ* was evaluated, a transect was established measuring 3.80 m, parallel to that described in Section 3.3.1. The distance between the transects of PR and *ρa* was 0.20 m. The measurements of PR were obtained on the same date as those of the *ρa*. Twenty (20) points were checked for PR along the transect at a distance of 0.20 m from one to another to the depth of 0.60 m. On an electronic spreadsheet, the PR data were clustered at each 0.05 m of depth. This made for a total of 240 values of PR per transect, resulting in 3600 values recorded.

#### 3.3.3. Bulk Density (Bd)

Bulk density (Bd) was determined on undisturbed soil samples in volumetric rings (0.025 m height by 0.06 m diameter) collected using an Uhland sampler [111] seven months after soil tillage. Four samples (replicates) were taken at a spacing of 0.20 m from one to another in the center of each layer (0–0.20, 0.20–0.40, and 0.40–0.60 m), taking the center of the planting furrow as a reference in the three replications of each treatment in each soil, for a total of 540 samples. In the laboratory, the samples were dried in a laboratory oven at 105–110 °C for 48 h to obtain soil dry mass (SDM), and Bd (g·cm^−3^) was calculated as the ratio between SDM and its volume.

The critical bulk density (Bdc) values based on soil texture were plotted together with the Bd values for comparison of the results. Critical bulk density refers to the variation in Bd values when the least limiting water range (LLWR) is zero (Bdc LLWR). The Bdc LLWR values were different for each soil texture class based on previous studies and compiled in Reichert et al. [89] in a pedotransfer function (Bdc LLWR = 0.00078 clay + 1.83803).

### 3.4. Statistical Analyses

The *ρ* values were exported from the Res2DINVDemo 4.9.17 (Geotomo Software, Penang, Malaysia) (Geotomo, 2017) after being inverted and filtered for the SURFER 13.6.618 software (Golden Software, Golden, CO, USA), just as the PR data for linear interpolation by triangulation, in order to obtain 2D maps for each treatment in each soil.

The Bd and VESS data (see Supplementary Materials) were tested regarding normality, independence, and homogeneity of variance. Upon meeting these presuppositions, analysis of variance (ANOVA) tests were carried out, and means were compared (Tukey’s test, *p* < 0.05) for each soil class. For ANOVA, a completely randomized design (CRD) was adopted, with four replications for Bd and five replications for VESS. For Bd, a mixed linear model was used to compare the depths within each soil class.

To evaluate the ability of ERT in detecting changes caused by soil management practices, Pearson linear correlation analysis was performed between *ρ*, PR, Bd, and the sampling depth. The PR and *ρ* data were extracted from a subset of data corresponding to the soil layer collected for analysis of Bd to compose the correlation matrix.

## 4. Conclusions

Our results showed that the soil response to different tillage systems and their effects on soil structure is dependent on soil class. Differently from most of the other papers, we demonstrated, by several aspects, the importance of soil class and soil structure on the effects of different tillage systems. Given their influence, we understand that soil classification and characterization of soil structure are crucial to better understand the dimension of the effects of different tillage systems.

The soil tillage strategies reduced resistance to penetration and increased electrical resistivity, as observed in the 2D maps. The greatest effect on soil structure, which led to better physical quality, was brought about by the treatments with deep tillage that mixed the soil in the 0–0.60 m layer (DT and DT + Ca), and ensured greater structural alleviation. This is best observed in the Inceptisol and in the Ultisol, in which the contrast between the areas mobilized and not mobilized by soil tillage was greater. In the Oxisol, due to its high structural quality, a natural condition brought about by its unique and stable microgranular structure, the effect of the treatments was not expressive, not overcoming the genetic control of the soil class. Therefore, deep tillage strategies should consider the morphogenetic conditions of the soil class in decision making processes for two reasons: (i) to avoid unnecessary tillage operations and (ii) to indicate operations for increasing soil quality by improving soil functions and ecosystem services.

The VESS did not show sensitivity to define differences among the soil tillage systems, probably due to the short time space between soil tillage and when VESS was carried out. Bulk density was not a good indicator of the structural changes caused by tillage strategies in the Rhodic Hapludult and the Rhodic Hapludox, since these soils tend to reconsolidate more quickly. Bulk density is therefore more sensitive in the Typic Dystrustept, a younger soil under tropical conditions. Soil electrical resistivity was positively correlated with bulk density throughout the dataset considering all the soil classes, and positively correlated with resistance to penetration in the Inceptisol. This study confirmed the potential for identification of structural changes caused by deep tillage in tropical soils on a field scale, and for characterization and monitoring of agricultural management practices. Future studies may concentrate on application of geophysical techniques to assist in the use of precise agriculture practices for efficient management of resources and increase in crop yield, which assists in decision making.

## Figures and Tables

**Table 1:** Physical characterization of the soils of the experimental area.

Soil		CX			PVd2			LVe2		
Horizon		A	Bw	BC	A	BA	Bt	A	Bo1	Bo2
Depth	m	0–0.05	0.05–0.15	0.15–0.60+	0–0.12	0.12–0.35	0.35–0.55+	0–0.10	0.10–0.60	0.60+
Bd	g cm^−3^	1.38	1.41	1.47	1.11	1.42	1.47	1.32	1.06	1.19
Pd		2.55	2.63	2.63	2.53	2.63	2.7	2.64	2.73	2.69
Tp	m^3^ m^−3^	0.49	0.45	0.48	0.54	0.46	0.46	0.54	0.57	0.53
FC		0.36	0.37	0.41	0.32	0.33	0.38	0.40	0.33	0.38
Mic		0.38	0.39	0.42	0.33	0.34	0.39	0.41	0.34	0.40
Mac		0.11	0.07	0.06	0.21	0.12	0.08	0.13	0.23	0.13
AC		0.25	0.17	0.15	0.41	0.28	0.18	0.26	0.43	0.28
Clay	%	41.7	35.5	35.5	44.8	46.9	67.3	50.6	65.5	68.6
Sand		41.9	28.4	28.4	40.6	38.9	24.0	29.6	21.3	20.1
Silt		16.5	36.1	36.1	14.5	14.2	8.64	19.7	13.1	11.3
Texture Class		Clay	Clay loam	Clay loam	Clay	Clay	Clay	Clay	Clay	Clay

CX: Typic Dystrustept; PV: Rhodic Hapludult; LV: Rhodic Hapludox. Bd: bulk density; Pd: particle density; Tp: total porosity; FC: field capacity estimated at −10 kPa; Mic: Microporosity estimated at −6 kPa; Mac: Macroporosity determined according to [47]; CA: soil aeration capacity determined according to [48]; Texture Class according to the Soil Survey Division [49].

**Table 2:** Summary of the parameters of inversion and root mean square (RMS).

Soil	Treatment	No. of Points Rejected	No. of Points Used	RMS %
CX	MT	13	148	15.7
	CT	45	116	15.8
	SB	10	151	14.6
	DM	19	142	14.5
	DM + Ca	14	147	14.0
PV	MT	12	149	14.3
	CT	3	158	9.0
	SB	4	157	14.5
	DM	3	158	14.1
	DM + Ca	11	150	14.7
LV	MT	4	157	15.2
	CT	2	159	14.5
	SB	6	155	15.5
	DM	5	156	9.0
	DM + Ca	3	158	10.7
Mean		10	151	13.7

Number of interations = 4; CX: Typic Dystrustept, PV: Rhodic Hapludult, and LV: Rhodic Hapludox; MT: minimum tillage with a pit (0.4 m in diameter by 0.7 m deep); CT: conventional tillage (0.25 m deep); SB: subsoiler with booted ripper points on two shanks (0.45 m deep); DM: deep mixing tillage with rotary hoe tiller (0.5 wide by 0.6 m deep); DM + Ca: deep mixing tillage with rotary hoe tiller (0.5 wide by 0.6 m deep) + additional liming.

**Table 3:** Chemical characterization of the soils of the experimental area.

Soil		CX			PV			LV		
Depth	m	0–0.2	0.2–0.4	0.4–0.6	0–0.2	0.2–0.4	0.4–0.6	0–0.2	0.2–0.4	0.4–0.6
pH	H_2_O	6.30	5.70	5.50	6.00	5.70	5.70	6.90	6.50	6.00
	CaCl_2_	5.70	5.10	4.90	5.40	5.10	5.10	6.30	5.90	5.40
P–Mehlich-1	mg kg^−1^	14.2	3.38	2.50	2.55	2.27	0.71	11.1	10.8	1.90
P–resin		25.4	5.73	1.18	10.9	5.25	3.41	10.4	4.76	3.45
K–Mehlich-1		94.0	45.4	32.5	133	103.0	71.7	66.5	38.9	23.4
Ca	cmol_c_ kg^−1^	5.42	3.38	2.50	4.22	3.12	2.41	4.76	2.86	2.14
Mg		1.25	0.59	0.44	1.11	0.85	0.57	1.19	0.95	0.60
Al		0.00	0.06	0.15	0.00	0.00	0.00	0.00	0.00	0.00
H + Al		3.06	4.12	3.68	2.55	3.41	3.55	1.79	2.14	2.86
CEC_7_		10.1	8.38	6.62	8.21	7.67	3.12	7.85	3.93	2.86
OC	%	1.40	0.90	0.90	1.80	1.10	0.80	1.10	1.10	0.60
SOM		2.41	1.55	1.55	3.10	1.90	1.38	1.90	1.90	1.03
BS		9.40	50.8	46.0	69.1	55.3	46.6	77.4	64.6	49.5
Al_sat_		0.00	1.30	3.50	0.00	0.00	0.00	0.00	0.00	0.00

CX: Typic Dystrustept; PV: Rhodic Hapludult; LV: Rhodic Hapludox. OC = organic carbon; SOM = soil organic matter; BS = Base saturation; Al_sat_ = Aluminum saturation.

## Data Availability

The data that support the findings of this study are available from the corresponding author upon reasonable request.

## Supplementary Materials

Data (Table S1) and discussion about the Visual Evaluation of Soil Structure (VESS) can be downloaded at: https://www.mdpi.com/article/10.3390/plants11172255/s1, Table S1: Visual evaluation of soil structure (VESS) under different soil tillage systems in three soil classes [112,113,114,115].

## References

[1] Rabot E., Wiesmeier M., Schlüter S., Vogel H.-J. (2018). Soil structure as an indicator of soil functions: A review. Geoderma.

[2] Hamilton G.J., Bakker D., Akbar G., Hassan Z., Mchugh A., Raine S. (2019). Deep blade loosening increases root growth, organic carbono, aeration, drainage, lateral infiltration and productivity. Geoderma.

[3] Souza L.S., Souza L.D., Paiva A.Q., Rodrigues A.C.V., Ribeiro L.S. (2008). Distribuição do sistema radicular de citros em uma topossequência de solos de tabuleiro costeiro do estado da Bahia. Rev. Bras. Cienc. Solo.

[4] Gao W., Hodgkinson L., Jin K., Watts C.W., Ashton R.W., Shen J., Ren T., Dodd I.C., Binley A., Phillips A.L. (2016). Deep roots and soil structure. Plant Cell Environ..

[5] Bengough A.G., Mckenzie B.M., Hallett P.D., Valentine T.A. (2011). Root elongation, water stress, and mechanical impedance: A review of limiting stresses and beneficial root tip traits. J. Exp. Bot..

[6] White R.G., Kirkegaard J.A. (2010). The distribution and abundance of wheat roots in a dense, structured subsoil—Implications for water uptake. Plant Cell Environ..

[7] Schneider F., Don A., Hennings I., Schmittmann O., Seidel S.J. (2017). The effect of deep tillage on crop yield—What do we really know?. Soil Tillage Res..

[8] Scanlan C.A., Davies S.L. (2019). Soil mixing and redistribution by strategic deep tillage in sandy soil. Soil Tillage Res..

[9] Barbosa S.M., Silva B.M., Oliveira G.C., Benevenute P.A.N., Silva R.F., Curi N., Moretti B.S., Silva S.H.G., Norton L.D., Pereira V.M. (2020). Deep furrow and additional liming for coffee cultivation under first year in a naturally dense inceptisol. Goederma.

[10] Kirkegaard J.A., Lilley J.M., Howe G.N., Graham J.M. (2007). Impact of subsoil water use on wheat yield. Aust. J. Agric. Res..

[11] Serafim M.E., Oliveira G.C., Lima J.M., Silva B.M., Zeviani W.M., Lima V.M.P. (2013). Disponibilidade hídrica e distinção de ambientes para cultivo de cafeeiros. Rev. Bras. Eng. Agríc. Ambient..

[12] Silva B.M., Oliveira G.C., Serafim M.E., Silva E.A., Ferreira M.M., Norton L.D., Curi N. (2015). Critical soil moisture range for a coffee crop in an oxidic Latosol as affected by soil management. Soil Tillage Res..

[13] Bünemann E.K., Bongiorno G., Bai Z., Creamer R.E., De Deyn G., Goede R., Fleskens L., Geissen V., Kuyper T.W., Mäder P. (2018). Soil quality—A critical review. Soil Biol. Biochem..

[14] Serafim M.E., Oliveira G.C., Oliveira A.S., Lima J.M., Guimarães P.T.G., Costa J.C. (2011). Sistema conservacionista e de manejo intensivo do solo no cultivo de cafeeiros na região do Alto São Francisco, MG: Um estudo de caso. Biosci. J..

[15] Serafim M.E., Oliveira G.C., Vitorino A.C.T., Silva B.M., Carducci C.E. (2013). Qualidade física e intervalo hídrico ótimo em Latossolo e Cambissolo, cultivados com cafeeiro, sob manejo conservacionista do solo. Rev. Bras. Cienc. Solo.

[16] Serafim M.E., Oliveira G.C., Curi N., Lima J.M., Guimarães P.T.G., Lima V.M.P. (2013). Potencialidades e limitações de uso de Latossolos e Cambissolos, sob sistema conservacionista em lavouras cafeeiras. Biosci. J..

[17] Santos W.J.R., Silva B.M., Oliveira G.C., Volpato M.M.L., Lima J.M., Curi N., Marques J.J. (2014). Soil moisture in the root zone and its relation to plant vigor assessed by remote sensing at management scale. Geoderma.

[18] Silva B.M., Santos W.J.R., Oliveira G.C., Lima J.M., Curi N., Marques J.J. (2015). Soil moisture space-time analysis to support improved crop management. Ciênc. Agrotecnol..

[19] Silva E.A., Oliveira G.C., Carducci C.E., Lima J.M., Melo L.B.B., Benevenute P.A.N. (2016). Stability of soil aggregates in Latosols and Cambisols via standard method and sonification. Afr. J. Agric. Res..

[20] Silva E.A., Carducci C.E., OliveirA G.C., Silva B.M., Serafim M.E. (2017). Estrutura de solos em manejo conservacionista: Diagnóstico visual, laboratorial, caracterização e inter-relações. Sci. Agrar..

[21] Oliveira G.C., Silva B.M., Carducci C.E., Silva S.H.G., Serafim M.E., Barbosa S.M., Silva E.A., Melo L.B.B., Benevenute P.A.N., Severiano E.C., Moraes M.F., Paula A.M. (2019). Melhoria físico-hídrica do ambiente radicular do cafeeiro em condições de sequeiro e implicações no uso da água. Tópicos em Ciência Do Solo.

[22] Silva B.M., Oliveira G.C., Serafim M.E., Silva E.A., Guimarães P.T.G., Melo L.B.B., Norton L.D., Curi N. (2019). Soil moisture associated with limiting water range, leaf water potential, initial growth and yield of coffee as affected by soil management system. Soil Tillage Res..

[23] Silva B.M., Oliveira G.C., Serafim M.E., Carducci C.E., Silva E.A., Barbosa S.M., Melo L.B.B., Santos W.J.R., Reis T.H.P., Oliveira C.H.C. (2019). Soil Management and Water-Use Efficiency in Brazilian Coffee Crops. Coffee-Prod. Res..

[24] Coulouma C., Boizard H., Trotoux G., Lagacherie P., Richard G. (2006). Effect of deep tillage for vineyard establishment on soil structure: A case study in Southern France. Soil Tillage Res..

[25] Bordoni M., Vercesi A., Maeker M., Ganimede C., Reguzzi M.C., Capelli E., Wei X., Mazzoni E., Simoni S., Gagnarli E. (2019). Effect of vineyard soil management on the characteristics of soils and roots in lower Oltrepò Apennines (Lombardy, Italy). Sci. Total Environ..

[26] Besson A., Cousin I., Samouëlian A., Boizard H., Richard G. (2004). Structural heterogeneity of the soil tilled layer as characterized by 2D electrical resistivity surveying. Soil Tillage Res..

[27] Séger M., Cousin I., Frison A., Boizard H., Richard G. (2009). Characterisation of the structural heterogeneity of the soil tilled layer by using in situ 2D and 3D electrical resistivity measurements. Soil Tillage Res..

[28] Besson A., Séger M., Giot A., Cousin I. (2013). Identifying the characristic scales of soil structural recovery after compaction from three in-field methods os monitoring. Geoderma.

[29] Loke M.H., Chambers J., Rucker D., Kuras O., Wilkinson P. (2013). Recent developments in the direct-current geoelectrical imaging method. J. Appl. Geophys..

[30] Rossi R., Amato M., Pollice A., Bitella G., Gomes J.J., Bochicchio R., Baronti S. (2013). Electrical resistivity tomography to detect the effects of tillage in a soil with a variable rock fragment content. Eur. J. Soil Sci..

[31] Kowalczyk S., Maślakowski M., Tucholka P. (2014). Determination of the correlation between the electrical resistivity of non-cohesive soils and the degree of compaction. J. Appl. Geophys..

[32] Jeřábek J., Zumr D., Dostál T. (2017). Identifying the plough pan position on cultivated soils by measurements of electrical resistivity and penetration resistance. Soil Tillage Res..

[33] Piccoli I., Furlan L., Lazzaro B., Morari F. (2020). Examining conservation agriculture soil profiles: Outcomes from northeastern Italian silty soils combining indirect geophysical and direct assessment methods. Eur. J. Soil Sci..

[34] Roodposhti H.R., Hafizi M.K., Kermani M.R.S., Nik M.R.G. (2019). Electrical resistivity method for water content and compaction evaluation, a laboratory test on construction material. J. Appl. Geophys..

[35] Melo L.B.B., Silva B.M., Peixoto D.S., Chiarini T.P.A., Oliveira G.C., Curi N. (2021). Effect of compaction on the relationship between electrical resistivity and soil water content in Oxisol. Soil Tillage Res..

[36] Vanella D., Cassiani G., Busato L., Boaga J., Barbagallo S., Binley A., Consoli S. (2018). Use of small scale electrical resistivity tomography to identify soil-root interactions during deficit irrigation. J. Hydrol..

[37] Banton O., Seguin M.K., Cimon M.A. (1997). Mapping field scale physical properties of soil with electrical resistivity. Soil Sci. Soc. Am. J..

[38] Samouëlian A., Cousin I., Tabbagh A., Bruand A., Richard G. (2005). Electrical resistivity survey in soil science: A review. Soil Tillage Res..

[39] Romero-Ruiz A., Linde N., Keller T., Or D. (2019). A review of geophysical methods for soil structure characterization. Rev. Geophys..

[40] Threadgill E.D. (1982). Residual tillage effects as determined by cone index. Trans. ASAE.

[41] Busscher W.J., Edwards J.H., Vepraskas M.J., Karlen D.L. (1995). Residual effects of slit tillage and subsoiling in a hardpan soil. Soil Tillage Res..

[42] Jonard F., Mahmoudzadeh M., Roisin C., Weihermüller L., André F., Minet J., Vereecken H., Lambot S. (2013). Characterization of tillage effects on the spatial variation of soil properties using ground-penetrating radar and electromagnetic induction. Geoderma.

[43] Bölenius E., Stenberg B., Arvidsson J. (2017). Within field cereal yield variability as affected by soil physical properties and weather variations—A case study in east central Sweden. Geoderma Reg..

[44] Sinnett D., Morgan G., Williams M., Hutchings T. (2008). Soil penetration resistance and tree root development. Soil Use Manag..

[45] Colombi T., Torres L.C., Walter A., Keller T. (2018). Feedbacks between soil penetration resistance, root architecture and water uptake limit water accessibility and crop growth—A vicious circle. Sci. Total Environ..

[46] Pereira T.T.C., Ker J.C., Shaefer C.E.G.R., Barros N.B., Neves J.C.L., Almeida C.C. (2010). Gênese de Latossolos e Cambissolos desenvolvidos de rochas pelíticas do grupo Bambuí—Minas Gerais. Rev. Bras. Cienc. Solo.

[47] Teixeira P.C., Donagemma G.K., Fontana A., Teixeira W.G. (2017). Manual de Métodos de Análise de Solo.

[48] Reynolds W.D., Drury C.F., Tan C.S., Fox C.A., Yang X.M. (2009). Use of indicators and pore volume-function characteristics to quantify soil physical quality. Geoderma.

[49] Soil Survey Division Staff (2017). Soil Survey Manual. Soil Conservation Service.

[50] Santos H.G., Jacomine P.K.T., Anjos L.H.C., Oliveira V.A., Lumbreras J.F., Coelho M.R., Almeida J.A., Filho J.C.A., Oliveira J.B., Cunha T.J.F. (2018). Sistema Brasileiro de Classificação de Solos.

[51] Ferreira M.M., Fernandes B., Curi N. (1999). Influência da mineralogia da fração argila nas propriedades físicas de Latossolos da região sudeste do Brasil. Rev. Bras. Cienc. Solo.

[52] Pereira T.T.C., Ker J.C., Almeida C.C. (2012). Qualidade de solos cultivados com eucalipto na região central de Minas Gerais: Atributos físicos, químicos e mineralógicos. Rev. Bras. Cienc. Agrar..

[53] Busscher W.J., Bauer P.J., Frederick J.R. (2002). Recompaction of a coastal loamy sand after deep tillage as a function of subsequent cumulative rainfall. Soil Tillage Res..

[54] Reichert J.M., Brandt A.A., Rodrigues M.F., Veiga M., Reinert D.J. (2017). Is chiseling or inverting tillage required to improve mechanical and hydraulic properties of sandy clay loam soil under long-term no-tillage?. Geoderma.

[55] García-Tomillo A., de Figueiredo T., Almeida A., Rodrigues J., Dafonte J.D., Paz-González A., Nunes J., Hernandez Z. (2017). Comparing effects of tillage treatments performed with animal traction on soil physical properties and soil electrical resistivity: Preliminary experimental results. Open Agric..

[56] Jayawickreme D.H., Van Dam R.L., Hyndman D.W. (2010). Hydrological consequences of land-cover change: Quantifying the influence of plants on soil moisture with time-lapse electrical resistivity. Geophysics.

[57] Loke M.H. Tutorial: 2-D and 3-D Electrical Imaging Surveys. http://www.geotomosoft.com/downloads.php.

[58] Srayeddin I., Doussan C. (2009). Estimation of the spatial variability of root water uptake of maize and sorghum at the field scale by electrical resistivity tomography. Plant Soil.

[59] Carminati A., Vetterlein D., Weller U., Vogel H.-J., Oswald S.E. (2009). When roots lose contact. Vadose Zone J..

[60] Bottraud J.C., Bornand M., Servat E. (1984). Mesures de résistivité et étude du comportement agronomique d’un sol. Bull. L’Assoc. Fr. L’Etude Sol.

[61] Zhou M., Wang J., Cai L., Fan Y., Zheng Z. (2015). Laboratoory investigations on factors affecting soil electrical resistivity and the measurement. IEEE Trans. Ind. Appl..

[62] Seladji S., Cosenza P., Tabbagh A., Ranger J., Richard G. (2010). The effect of compaction on soil electrical resistivity: A laboratory investigation. Eur. J. Soil Sci..

[63] Filho O.J.V., Souza Z.M., Silva R.B., Lima C.C., Pereira D.M.G., Lima M.E., Sousa A.C.M., Souza G.S. (2015). Capacidade de suporte de carga de Latossolo Vermelho cultivado com cana-de-açúcar e efeitos da mecanização no solo. Pesq. Agropec. Bras..

[64] Soil Survey Staff (2014). Keys to Soil Taxonomy.

[65] McCarter W.J. (1984). The electrical resistivity characteristics of compacted clays. Géotechnique.

[66] García-Tomillo A., De Figueiredo T., Dafonte J.D., Almeida A., Paz-González A. (2018). Effects of machinery trafficking in an agricultural soil assessed by Electrical Resistivity Tomography (ERT). Open Agric..

[67] Naderi-Boldaji M., Sharifi A., Hemmat A., Alimardani R., Keller T. (2014). Feasibility study on the potential of electrical conductivity sensor Veris^®^ 3100 for field mapping of topsoil strength. Biosyst. Eng..

[68] Lipiec J., Hatano R. (2003). Quantification of compaction effects on soil physical properties and crop growth. Geoderma.

[69] Imhoff S., Silva A.P., Fallow D. (2004). Susceptibility to compaction, load support capacity, and soil compressibility of Hapludox. Soil Sci. Soc. Am..

[70] Pott L.P., Amado T.J.C., Leal O.A., Ciampitti I.A. (2019). Mitigation of soil compaction for boosting crop productivity at varying yield environments in southern Brazil. Eur. J. Soil Sci..

[71] Etana A., Holm L., Rydberg T., Keller T. (2020). Soil and crop responses to controlled traffic farming in reduced tillage and no-till: Some experiences from field experiments and on-farm studies in Sweden. Acta Agric. Scand. Sect. B Soil Plant Sci..

[72] Reichert J.M., Reinert J.M., Braida J.A. (2003). Qualidade dos solos e sustentabilidade de sistemas agrícolas. Ciênc. ambient..

[73] Dexter A.R., Horn R., Holloway R., Jakobsen B.F. (1988). Pressure transmission beneath wheels in soils on the Eyre peninsula of South Australia. J. Terramech..

[74] Moraes M.T., Debiasi H., Carlesso R., Franchini J.C., Silva V.R., Luz F.B. (2017). Age-hardening phenomena in an oxisol from the subtropical region of Brazil. Soil Tillage Res..

[75] Utomo W.H., Dexter A.R. (1981). Age hardening of agricultural top soils. J. Soil Sci..

[76] Simões R.P., Raper R.L., Arriaga F.J., Balkcom K.S., Shaw J.N. (2009). Using conservation systems to alleviate soil compaction in a Southeastern United States ultisol. Soil Tillage Res..

[77] Hillel D. (2003). Introduction to Environmental Soil Physics.

[78] Drescher M.S., Eltz F.L.F., Denardin J.E., Faganello A. (2011). Persistência do efeito de intervenções mecânicas para a descompactação de solos sob plantio direto. Rev. Bras. Cienc. Solo.

[79] Bonetti A.J., Anghinoni I., Moraes M.T., Fink J.R. (2017). Resilience of soils with different texture, mineralogy and organic matter under long-term conservation systems. Soil Tillage Res..

[80] Reichert J.M., Kaiser D.R., Reinert D.J., Riquelme U.F.B. (2009). Variação temporal de propriedades físicas do solo e crescimento radicular de feijoeiro em quatro sistemas de manejo. Pesq. Agropec. Bras..

[81] Drescher M.S., Eltz F.L.F., Denardin J.E., Faganello A., Drescher G.L. (2012). Resistência à penetração e rendimento da soja após intervenção mecânica em Latossolo Vermelho sob plantio direto. Rev. Bras. Cienc. Solo.

[82] Nicoloso R.D.S., Amado T.J.C., Schneider S., Lanzanova M.E., Girardello V.C., Bragagnolo J. (2008). Eficiência da escarificação mecânica e biológica na melhoria dos atributos físicos de um Latossolo muito argiloso e no incremento do rendimento de soja. Rev. Bras. Cienc. Solo.

[83] Scarpare F.V., Van Lier Q.J., Camargo L., Pires R.C.M., Ruiz Corrêa S.T., Bezerra A.H.F., Gava G.J.C., Dias C.J.C. (2019). Tillage effects on soil physical condition and root growth associated with sugarcane water availability. Soil Tillage Res..

[84] Bavoso M.A., Silva A.P.D., Figueiredo G.C., Tormena C.A., Giarola N.F.B. (2012). Resiliência física de dois Latossolos vermelhos sob plantio direto. Rev. Bras. Cienc. Solo.

[85] Peixoto D.S., Silva B.M., Silva S.H.G., Karlen D.L., Moreira S.G., Silva A.A.P., Resende A.V., Norton L.D. (2019). Diagnosing, Ameliorating, and Monitoring Soil Compaction in No-Till Brazilian Soils. Agrosyst. Geosci. Environ..

[86] Abreu S.L., Reichert J.M., Reinert D.J. (2004). Escarificação mecânica e biológica para a redução da compactação em Argissolo franco-arenoso sob plantio direto. Rev. Bras. Cienc. Solo.

[87] Borchet H. (1984). Grenzen und Vorhersage der Bodenmeliorationswirkung bei der Tieflockerung. Mitteilungen der Dmsch. Bodenkd. Ges..

[88] Borchet H., Graf R. (1985). Über die Entwicklungstendez des Bodengefüges in tiefgelockerten Böden aus verschiedenen geologischen Substraten. Schr. Dtsch. Verb. Wasserwirtsch. Kult. e.V. (DVWK).

[89] Reichert J.M., Suzuki L.E.A.S., Reinert D.J., Horn R., Håkansson I. (2009). Reference bulk density and critical degree-of-compactness for no-till crop production in subtropical highly weathered soils. Soil Tillage Res..

[90] Busscher W.J., Bauer P.J., Frederick J.R. (2006). Deep tillage management for high strength southeastern USA Coastal Plain soils. Soil Tillage Res..

[91] Busscher W.J., Khalilian A., Jones M.A. (2012). Tillage Management for Cotton in Southeastern Coastal Soils during Dry Years. Commun. Soil Sci. Plant Anal..

[92] Peixoto D.S., Silva B.M., Oliveira G.C., Moreira S.G., Silva F., Curi N. (2019). A soil compaction diagnosis method for occasional tillage recommendation under continuous no tillage system in Brazil. Soil Tillage Res..

[93] Basso B., Amato M., Bitella G., Rossi R., Kravchenko A., Sartori L., Carvahlo L.M., Gomes J. (2010). Two-dimensional spatial and temporal variation of soil physical properties in tillage systems using electrical resistivity tomography. Agron. J..

[94] Vaz C.M.P., Manieri J.M., Maria I.C., Tuller M. (2011). Modeling and correction of soil penetration resistance for varying soil water content. Geoderma.

[95] Vepraskas M.J., Busscher W.J., Edwards J.H. (1995). Residual effects of deep tillage vs. no-till on corn root growth and grain yield. J. Prod. Agric..

[96] Alvares C.A., Stape J.L., Sentelhas P.C., Gonçalves J.L.M., Sparovek G. (2013). Köppen’s climate classification map for Brazil. Meteorol. Z..

[97] INMET—Instituto Nacional de Meteorologia. http://www.inmet.gov.br/portal/.

[98] Andrade H., Alves H.M.R., Vieira T.G.C., Resende R.J.T.P., Esteves D.R., Brasil J.P.K., Rosa E.R. (1998). Diagnóstico ambiental do município de Lavras com base em dados georreferenciados do meio físico: IV—Principais grupamentos de solos. Proceedings of the Congresso Brasileiro de Engenharia Agrícola.

[99] Lacerda M.P.C., Andrade H., Quéméneur J.J.G. (2000). Micropedologia da alteração em perfis de solos com B textural na região de Lavras, Minas Gerais. Rev. Bras. Cienc. Solo.

[100] Curi N., Silva E., Gomes F.H., Menezes M.D., Silva S.H.G., Teixeira A.F.S. (2020). Mapeamento de Solos, Aptidão Agrícola e Taxa de Adequação do Uso das Terras do Município de Lavras (MG).

[101] Curi N., Silva S.H.G., Poggere G.C., Menezes M.D. (2017). Mapeamento de Solos e Magnetismo no Campus da UFLA como Traçadores Ambientais.

[102] Santos H.G., Carvalho Júnior W., Dart R.O., Áglio M.L.D., Sousa J.S., Pares J.G., Fontana A., Martins A.L.S., Oliveira A.P. (2011). O Novo Mapa de Solos do Brasil.

[103] Sawe B.E. USDA Soil Taxonomy: Soil Orders and Their Major Characteristics. https://www.worldatlas.com/articles/usda-soil-taxonomy-soil-orders-and-their-major-characteristics.html.

[104] Mafes (2017). BigMix AS-2, Preparador de Solo. http://mafes.com.br/big_mix.html.

[105] Rozane D.E., Natale W. (2014). Calagem, adubação e nutrição mineral de anonáceas. Rev. Bras. Frutic..

[106] Geotomo Software Rapid 2-D Resistivity & IP Inversion Using the Least Squares Methods. http://www.geotomosoft.com/downloads.php.

[107] Binley A., Kemna A., Rubin Y., Hubbard S.S. (2005). DC Resistivity and Induced Polarization Methods. Hydrogeophysics.

[108] Loke M.H., Barker R.D. (1996). Rapid least-squares inversion of apparent resistivity pseudosections using a quasi-Newton method. Geophys. Prospect..

[109] Stolf R. (1991). Teoria e teste experimental de fórmulas de transformação dos dados de penetrômetro de impacto em resistência do solo. Rev. Bras. Cienc. Solo.

[110] (2010). Soil Cone Penetrometer.

[111] Blake G.R., Hartge. K.H., Klute A. (1986). Bulk density. Methods of Soil Analysis: Part 1. Physical and Mineralogical Methods.

[112] Ball B.C., Batey T., Munkholm L.J. (2007). Field assessment of soil structural quality—A development of the Peerlkamp test. Soil Use Manag..

[113] Kondo M.K., Dias Júnior M.S. (1999). Compressibilidade de três Latossolos em função da umidade e uso. Rev. Bras. Cienc. Solo.

[114] Bodner G., Leitner D., Kaul H.P. (2014). Coarse and fine root plants affect pore size distributions differently. Plant Soil.

[115] Guimarães R.M.L., Ball B.C., Tormena C.A. (2011). Improvements in the visual evaluation of soil structure. Soil Use Manag..

