# Supplementary material for: Deep Tillage Strategies in Perennial Crop Installation: Structural Changes in Contrasting Soil Classes
Source: Plants (Basel). 2022 Aug 30;11(17):2255. doi: 10.3390/plants11172255 (PMC9460178; doi:10.3390/plants11172255)
Supplement: Supplementary file 1 [file plants-11-02255-s001.zip › plants-1809975-supplementary.pdf]

plants MDPI

Supplementary Materials

Deep Tillage Strategies in Perennial Crop Installation: Structural Changes in Contrasting Soil Classes

Results and Discussion

Visual Evaluation of Soil Structure (VESS)

Regardless of the tillage method and the class of soil, there were no differences in the VESS scores, except for MT, which exhibited scores in the following order for the soil classes: PV > LV > CX, in regard to physical quality (Table 4). In relation to the treatments, there were no differences in PV, whereas for LV and CX, the MT had a lower result for soil quality. In spite of that, scores of 1 and 2 are classified as "good" and do not require intensive management [112]. It should be noted that even though soil turnover did not occur in the MT treatment, there was surface furrowing (0.10 m) and boring of the plant hole, which cause rupture of the structure and mixture of the soil horizons.

The absence of differences in relation to soil tillage systems CT, SB, DT, and DT+Ca– is directly related not only to the implement used, but also to the plowing and disking that were performed beforehand, creating uniformity in the 0-0.25 m layer, the working depth of the plow, and eliminating the stress history [113] in all the soil classes. It is important to emphasize that the soil classes in this study were under fallow conditions for at least 5 years up to the time tillage was performed; they did not exhibit restrictions regarding fertility and had little mechanical disturbance, which allowed establishment of plant cover. At the time analysis was performed, PV was the soil with the greatest density and vigor of spontaneous vegetation, with predominance of *Brachiaria decumbens*, just as in CX, though with much lower vigor and density. In LV, *Panicum maximum* predominated. It should be highlighted that the presence of grass roots favors the formation and maintenance of macropores, with heterogenization of the porous space that is related to the growth of fine roots [114], which can explain the differences in the MT score (Table 4).

Table S1. Visual evaluation of soil structure (VESS) under different soil tillage systems in three soil classes.

Treatments	CX	PV	LV
Minimum-till+Pit – MT	1.74 c B	1.08 a A	1.33 b B
Conventional – CT	1.17 a A	1.09 a A	1.10 a A
Subsoiling - SB	1.00 a A	1.05 a A	1.06 a A
DeepMixing - DM	1.02 a A	1.00 a A	1.07 a A
DeepMixing+Ca - DM+Ca	1.00 a A	1.00 a A	1.00 a A

Mean values followed by the same lowercase letters in the row and uppercase letters in the column do not differ from each other by Tukey's test ($p < 0.05$). CX: Typic Dystrustept, PV: Rhodic Hapludult, and LV: Rhodic Hapludox.

The three soil classes evaluated in the study [101] have differences in the degree of pedogenesis, which is reflected in their intrinsic attributes, such as soil depth, structure, and density. These natural properties should be considered when carrying out VESS. The attributes intrinsic to the very clayey Oxisols (Latossolos) of tropical regions may become a confounding factor for the VESS. Its greater gibbsite content promotes good aggregation and high macroporosity associated to its very stable microgranular structure [51]. Ultisols (Argissolos) and Inceptisols (Cambissolos), with angular and subangular block structure

in this study, respectively, tend to break down into larger particles and with greater difficulty. These are characteristics that can directly affect evaluation in VESS, without necessarily compromising the soil functions and ecosystem services of these soil classes.

Materials and Methods

Visual Evaluation of Soil Structure (VESS)

The soil structure was visually evaluated according to the Visual Evaluation of Soil Structure (VESS) method developed by Ball et al. [112] and adapted by Guimarães et al. [115]. Soil blocks of 0.25 m depth, 0.10 m thickness, and 0.20 m width were collected from the center of the plant row of each experimental plot (n = 75), four months after soil tillage. The structure was evaluated, attributing scores from 1 (good soil structural quality) to 5 (poor structural quality) according to the criteria available in Guimarães et al. [115].
